# Endogenous estrogen metabolites as oxidative stress mediators and endometrial cancer biomarkers

**DOI:** 10.1186/s12964-024-01583-0

**Published:** 2024-04-02

**Authors:** Katarzyna Bukato, Tomasz Kostrzewa, Antonella Marino Gammazza, Magdalena Gorska-Ponikowska, Sambor Sawicki

**Affiliations:** 1 Department of Obstetrics and Gynecology, Oncological Gynecology and Gynecological Endocrinology, Medical University of Gdansk, Smoluchowskiego 17, Gdańsk, 80-214 Poland; 2 Department of Medical Chemistry, Faculty of Medicine, Medical University of Gdansk, Dębinki 1, Gdansk, 80-211 Poland; 3 Department of Biomedicine, Neuroscience and Advanced Diagnostics, University of Palermo, Palermo, 90127 Italy; 4 IEMEST Istituto Euro-Mediterraneo di Scienza e Tecnologia, Palermo, 90127 Italy; 5 Department of Biophysics, Institute of Biomaterials and Biomolecular Systems, University of Stuttgart, 70174 Stuttgart, Germany

**Keywords:** Biomarker, Endometrial cancer, Estradiol metabolites, 2-methoxyestradiol, Oxidative stress

## Abstract

**Background:**

Endometrial cancer is the most common gynecologic malignancy found in developed countries. Because therapy can be curative at first, early detection and diagnosis are crucial for successful treatment. Early diagnosis allows patients to avoid radical therapies and offers conservative management options. There are currently no proven biomarkers that predict the risk of disease occurrence, enable early identification or support prognostic evaluation. Consequently, there is increasing interest in discovering sensitive and specific biomarkers for the detection of endometrial cancer using noninvasive approaches.

**Content:**

Hormonal imbalance caused by unopposed estrogen affects the expression of genes involved in cell proliferation and apoptosis, which can lead to uncontrolled cell growth and carcinogenesis. In addition, due to their ability to cause oxidative stress, estradiol metabolites have both carcinogenic and anticarcinogenic properties. Catechol estrogens are converted to reactive quinones, resulting in oxidative DNA damage that can initiate the carcinogenic process. The molecular anticancer mechanisms are still not fully understood, but it has been established that some estradiol metabolites generate reactive oxygen species and reactive nitrogen species, resulting in nitro-oxidative stress that causes cancer cell cycle arrest or cell death. Therefore, identifying biomarkers that reflect this hormonal imbalance and the presence of endometrial cancer in minimally invasive or noninvasive samples such as blood or urine could significantly improve early detection and treatment outcomes.

## Abstract (summary)

This review analyzes the role of estrogen metabolites as potential biomarkers for the early detection and monitoring of endometrial cancer.

## Background

Exploring the metabolic pathway of estradiol (E2) and the properties of individual metabolites may revolutionize the approach to diagnosing and treating the most prevalent gynecological cancer in high-income countries, endometrial cancer. Endometrial cancer is the fourth most prevalent cancer in women [1] and its incidence has risen in subsequent decades and successive generations in numerous countries, particularly those undergoing rapid socioeconomic transitions [1]. Postmenopausal bleeding (PMB) is the most common red-flag symptom of endometrial cancer [2]. According to the findings of a systematic review and meta-analysis, PMB has a high degree of sensitivity for the identification of endometrial cancer, because it is present in approximately 90% of cases [3]. However, most women with PMB are not diagnosed with endometrial cancer. Due to the low specificity of PMB, only about 9% of women ho have experienced bleeding will receive this diagnosis [4]. Despite the improvements that have been made in the diagnosis of cancer over the last few decades, diagnostic workup for endometrial cancer has experienced very little advancement, if any. Most endometrial cancers are diagnosed at a localized stage and are curable in most patients after surgery, with an approximately 95% 5-year survival rate. In contrast, the 5-year survival rate for advanced endometrial cancer (stage IV) ranges from 16 to 45% [3]. Therefore, early detection of endometrial cancer is crucial to improving survival rates and reducing the need for aggressive treatments.

## Content

### Current diagnostic standards

**Fig. 1:**
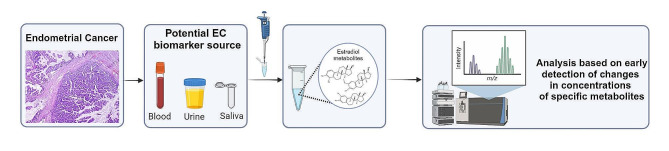
Diagnostic path for patients suspected of endometrial cancer using noninvasive methods

Transvaginal ultrasonography is commonly implemented in the differential diagnosis of abnormal uterine bleeding. Many guidelines propose transvaginal ultrasonography assessment of endometrial thickness (ET) as a noninvasive first-line examination to forecast the risk of endometrial cancer based on the correlation between endometrial cancer and ET [3]. The ET is used as a risk indicator of endometrial cancer and determines whether further, invasive investigations, such as endometrial biopsy, are necessary [1]. There are numerous ET cutoff values that necessitate surgical intervention in patients with PMB. According to meta-analyses, a typical 98% sensitivity, cutoff value of ET for preventing endometrial sampling in nearly half of women with PMB is 3 mm [5, 6]. However, all ET cutoff values with sensitivity rates above 90% (1 to 5 mm) had false positive rates of 70% or more [7]. As there is no threshold value that combines high sensitivity with clinically acceptable low false-positive rates, ET measured by transvaginal ultrasonography shows limited usefulness as a diagnostic tool for predicting the presence of endometrial cancerin women with PMB [7]. Therefore, other factors such as patient age, risk factors for endometrial cancer, and the presence of abnormal uterine bleeding should be considered when determiningthe need for surgical intervention in women with PMB. Endometrial tissue sampling and subsequent histological testing provide the most precise diagnosis. Dilatation and curettage and pipelle biopsies are the most frequently used procedures. All outpatient endometrial sampling techniques have limited patient acceptability due to pain, discomfort, or access problems caused by cervical stenosis or atrophy. In addition, invasive procedure can lead to infection, uncommon uterine perforation, or other complications, and may result in bleeding. Even with hysteroscopy and endometrial curettage, only 65% of the endometrial cavity can be sampled, which can cause sampling error. The concordance between endometrial biopsy and hysterectomy for all histological findings is between 60% and 70% [1]. Following a failed endometrial sample or in situations of an irregular endometrium, in the presence of other risk factors, hysteroscopy with targeted biopsy is necessary, however, this is an invasive procedure and sometimes associated with operative difficulties [8]. Currently, standard diagnostic procedures for endometrial cancer can be uncomfortable, expensive, painful, and even dangerous, especially for nulliparous women [2]. The poor positive predictive value of PMB highlights the need for new triage tests with high specificity to optimize PMB management [3]. An endometrial cancer detection tool that can accurately identify women who require invasive testing would significantly improve clinical practice in patients with PMB. This approach would not only save women from unnecessary pain and discomfort but also optimize PMB management and prevent thousands of unnecessary invasive tests. A more streamlined approach to PMB management could have significant benefits for both patients and healthcare providers, leading to better health outcomes and reduced healthcare costs over time. One potential solution is the development of more accurate diagnostic tools that can quickly and easily identify the underlying cause of PMB. This could involve the use of biomarkers that can be detected through blood tests or noninvasive methods (Fig. 1). E2 metabolites are already acknowledged as indicators of oxidative stress [9], which plays a crucial role in carcinogenesis and could be used to find endometrial cancer-specific biomarkers.

### Estrogen metabolic pathways

**Fig. 2:**
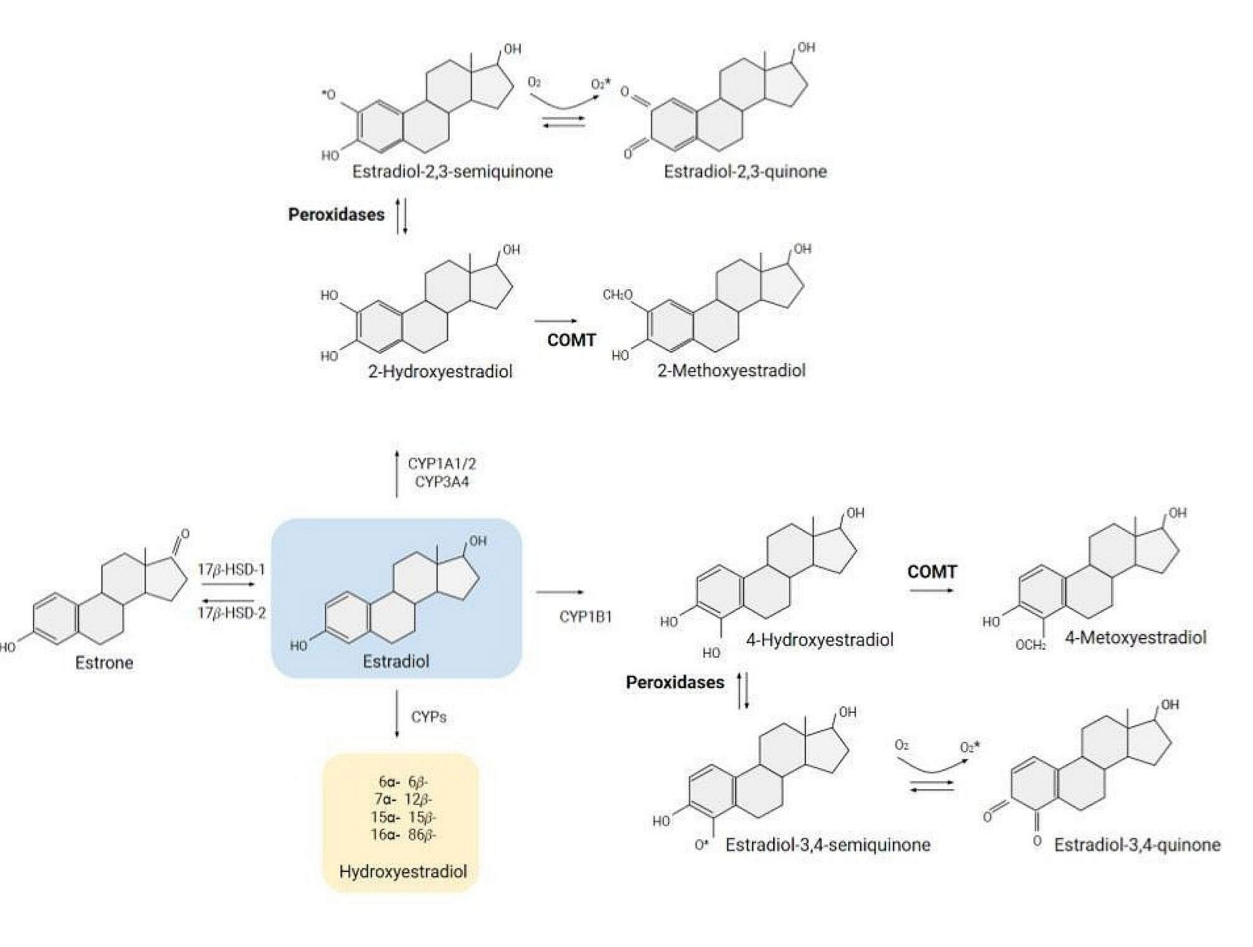
Metabolic pathway of estradiol

The best documented biochemical finding associated with endometrial carcinogenesis is a hormonal imbalance caused by unopposed estrogen [10], which is a key contributor to each of the established risk factors for this disease, including obesity, early menarche, late menopause, nulliparity, menopausal hormone use, polycystic ovarian syndrome, and tamoxifen use [10, 11]. In addition, some data suggest that endometrial tumor tissues are characterized by an imbalance of various biotransformation pathways, which causes increased levels of circulating estrogen in patients with endometrial cancer [12]. Circulating estrogen levels are greater in women with endometrial cancer than in healthy postmenopausal individuals [13, 14]. The levels of E2 and estrone (E1) are considerably greater in patients with uterine cancer (1.4- and 1.5-fold, respectively) [15]. Zeleniuch-Jacquotte et al. reported a similar trend, demonstrating 2.4- and 3.9-fold increases in risk in the highest tertiles of the E2 and E1 quartiles, respectively [16]. Before analyzing the possibility of using estrogen metabolites as endometrial cancer biomarkers, it is important to understand the metabolic pathway of E2 and the effect of its metabolites on the carcinogenesis process (Fig. 2).

Estrogens are a class of C18 steroids characterized by the presence of a benzene ring, a phenolic hydroxyl group at C3, and either a hydroxyl group (17β-estradiol) or a ketone group E1 at C17 [17]. E2 is widely recognized as the most biologically active form of estrogen, the main precursor to which is E1 [18]. E1 exists in two forms: unbound E1 and E1-sulfate (E1-S). The enzyme steroid sulfatase converts E1-S into active (free) E1, which is then transported across the cell membrane by specific transport proteins [18]. Intracellular E1 can be converted back to E1-S by estrogen sulfotransferase [19]. The serum level of E1-S in women with endometrial cancer can be 4.5 times higher than that in healthy women (*p* < 0.001) [20]. Another pathway of action of E1 is its reversible conversion to E2, which is facilitated by the enzyme activity of 17-hydroxysteroid dehydrogenase [21]. In particular, the enzyme HSD17β type 1 promotes the process of 17β-reduction, converting E1, which has low biological activity, into the highly potent E2. On the other hand, HSD17β type 2 preferentially catalyzes the oxidation of E2 back to E1 [22]. These enzymes are important for maintaining the balance of estrogen levels and regulating the effects of estrogen in tissues. In healthy tissues, this balance favors E1 formation [23]. Dysregulation of HSD17 activity can contribute to the development of estrogen-related disorders such as endometrial hyperplasia and cancer [24, 25] The activity ratio of reducing to oxidizing HSD17β is greater in low-grade endometrial cancerthan in healthy postmenopausal endometrium and peritumoral tissues [23]. One hypothesis posits that the upregulation of HSD17β1 results in enhanced estrogen action at target tissues via an increase in the E2/E1 ratio and a higher concentration of highly active ligands for estrogen receptors (ERs). Both pre- and postmenopausal tissues exhibit oxidative and reductive HSD17 activities [26]. However, the presence of HSD17β1 in the human endometrium and in endometrial cancer is controversial. Several studies have reported that in endometrial cancer tissues, HSD17β1 expression and enzyme activity are increased compared with those in normal endometrium [13, 23]. Another study showed a notable decrease in expression [27]. In endometrial adenocarcinoma, HSD17β type 1 expression and activity are not always detected [25, 28]. Research conducted on mice has demonstrated that the excessive production of human HSD17β1 intensifies the effects of estrogen in the uterus, leading to the development of endometrial hyperplasia, both with and without atypia. Nevertheless, in the present study, HSD17β1 did not cause endometrial carcinoma, suggesting that other mechanisms, such as inactivation of phosphatase and tensin homolog, loss of forkhead box O subclass transcription factor 1, and hyperactivity of the phosphoinositide-3 kinase pathway, play crucial roles in the development of endometrial cancer [24]. Additionally, other estrogen-metabolizing enzymes, such as HSD17β7, HSD17β12, HSD17β5, HSD17β2, HSD17β4, HSD17β8, aromatase, steroid sulfatase, and estrogen sulfotransferase determine the hormonal status of the endometrium [24]. One possible explanations for these inconsistencies is the limited sensitivity of conventional detection methods due to the low expression of HSD17β1 [26]. HSD17β type 2 activity is detected in most endometrial hyperplasia cases and less than half of carcinoma cases [25]. Data show that in premenopausal patients, HSD17β2 inactivates E2 in situ, acting as a part of a protective and/or suppressive mechanism against unopposed estrogenic effects [25]. In contrast to premenopausal women, in postmenopausal patients HSD17β type 2 may not play important roles because of the low availability of in situ E2 in endometrial carcinoma tissue [25]. Moreover, HSD17 maintains an equilibrium between hydroxy- and methoxyestrone and E2 metabolites, primarily in the liver, prior to further metabolism and elimination [29]. However, further research is needed to understand the role of intratumoral estrogen metabolism and biosynthesis in relation to HSD17. These findings will help clarify its potential as a biomarker of endometrial carcinoma. The first phase of estrogen metabolism is hydroxylation [22]. Estrogen metabolites perform additional biological functions as a result of the metabolic conversion of estrogens by three competitive pathways involving irreversible hydroxylation catalyzed by NADPH-dependent cytochrome P450 (CYP) enzymes, including CYP1A1, CYP1B1, and CYP1A2 [12]. E1 and E2 undergo hydroxylation at positions C2, C4, and C16, resulting in the formation of catechol estrogens [30]. They can undergo hydroxylation via two distinct pathways, namely, the hydroxylation of the A-ring or the D-ring. A-ring metabolism produces the catechol estrogens: 2-hydroxyestradiol (2-OH-E2), 2-hydroxyestrone (2-OH-E1), 4-hydroxyestradiol (4-OH-E2), and 4-hydroxyestrone (4-OH-E1), whereas D-ring metabolism produces 16-hydroxyestrone (16-OH-E1) and estriol [13, 21]. Studies on the pro- and anticarcinogenic properties of different E2 metabolites are ongoing, and these studies may explain the notable variations in the metabolite concentrations in the tissues and bodily fluids of patients with endometrial cancer. There are still many contradictory results among the studies conducted thus far. However, some of them agree on the carcinogenic effects of estriol, 16-hydroxyestrone, and the protective qualities of 2-methoxyestradiol [31–33]. As a result, a shift in the metabolic pathways of the A- and D- rings in favor of the D-ring is regarded by some as a biological marker of cancer risk [31]. Further research has suggested that patients with endometrial cancer exhibit elevated levels of each parent estrogen and majority metabolite. Catechol estrogens in the 2-pathway and 4-pathway and 16α-hydroxyestrone are elevated among patients compared with controls [34, 35]. A prospective study comprising 124 patients assessed the levels of circulating 2-OHE1 and 16α-OHE1 and revealed some increases with high levels of both metabolites, but this relationship did not persist after adjustment for E1 or E2 levels [36]. These results do not support the hypothesis that greater metabolism of estrogen via the 2-OH pathway, relative to the 16*α*-OH pathway, protects against endometrial cancer [36]. The following phase of metabolic processes involves methylation. Catechol estrogens undergo additional methylation through the action of the catechol-O-methyltransferase (COMT) enzyme, resulting in the formation of methoxyestrogens such as 2-methoxyestrone (2-MeO-E1), 4-methoxyestrone (4-MeO-E1), 2-methoxyestradiol (2-MeO-E1, and 4-methoxyestradiol (4-MeO-E2) [22]. Estrogen can undergo deactivation and subsequent elimination from the body through additional mechanisms. Specifically, parent estrogens and catechol estrogens are conjugated with glucuronic acid and sulfate, facilitated by enzymes such as UDP-glucuronosyltransferases and sulfotransferases, respectively [37]. The mechanisms involved in the carcinogenic effects of estrogens continue to be a topic of interest. The conventional mechanism of estrogen’s carcinogenic activity relies on its interaction with ERs. The process of estrogen signaling is facilitated by two nuclear ERs namely ERα and ERβ, as well as one membrane receptor known as the G- proteincoupled ER The predominant receptor in endometrial tissue is ERα [38]. The ERs enable both genomic and nongenomic effects of estrogens upon binding to ligands [18]. The term “genomic signaling” pertains to the process by which the ER executes its conventional function as a steroid hormone receptor, leading to the binding of the ER to estrogen response elements located within genes that regulate cell proliferation. Nongenomic signaling involves the binding of the ER to the cell surface. Upon binding with estrogens, the ER triggers the activation of various signaling pathways [10, 39]. Numerous signaling pathways, including the PI3K/AKT/mTOR signaling pathway, WNT/β-catenin signal transduction cascades (including the APC/β-catenin pathway), the MAPK/ERK pathway, the VEGF/VEGFR ligand receptor signaling pathway, the ErbB signaling pathway, the P53/P21 signaling pathway and the P16INK4a/pRB signaling pathway, have been shown to be involved in the multiple steps of endometrial cancer development [40]. The scope of this review does not allow for a comprehensive consideration of these pathways. Consequently, increases in estrogen activity or exposure to exogenous estrogens may result in increased cell proliferation. It is hypothesized that swiftly proliferating cells are prone to errors because they have less time for DNA proofreading and repair. Incorporating errors into essential genes, such as oncogenes and/or tumor suppressors, is likely to result in mutations and malignant growth.

### Endogenous estrogen metabolites as oxidative stress mediators

There is evidence to support the theory that estrogen-induced tumors result from the redox cycling of estrogen metabolites [41]. Estrogens and their catecholic metabolites have been reported to act as prooxidants [41, 42]. The harmful effects of these metabolites can be associated either directly by making semiquinone radicals or indirectly as a result of their ability to undergo a redox cycle and form reactive oxygen species ROS [13, 42, 43]. 2-Hydroxyestradiol, a significant E2 metabolite, is mostly catalyzed by CYP1A2 and CYP3A4 in the liver and by CYP1A1 in extrahepatic tissues [44]. However, CYP1B1 is widely expressed in estrogen target tissues such as the breast, ovary, and uterus [44]. Immunohistochemical staining of endometrial carcinomas showed that CYP1B1 is upregulated in endometrial cancers [45]. The enzyme CYP1B1 plays a crucial role in the enzymatic hydroxylation of estrogens, leading to the formation of 4-hydroxyestrogens. The amount of 4-OH-E2 in the urine of patients with endometrial cancer was substantially greater than that in the control group, indicating a relationship between 4-OH-E2 and endometrial cancer occurrence [13]. An increased level of 4-hydroxyestradiol promotes both cell proliferation and metastasis [13]. The process of metabolically converting estrogens into 4-hydroxy estrogens has been hypothesized to have a significant impact on the development of cancer, as it can lead to DNA damage [45, 46]. The impact of CYP1B1 on the progression of the cell cycle in endometrial cancer cells and its role in regulating the expression of various genes associated with the cell cycle have been observed [45]. Catechol estrogens, specifically 4-OH-E2, can undergo redox cycling via nonenzymatic autoxidation to generate reactive semiquinone and quinone intermediates and contribute to the production of ROS This redox reaction of catechol estrogens is enhanced in the presence of Cu2 + or Fe3 + and by enzymatic catalysis by cytochrome P450 oxidases or peroxidases, accompanied by an increase in ROS production [37]. Redox cycling between o-quinones and their semiquinone radicals is thought to generate superoxide, hydrogen peroxide, and reactive hydroxyl radicals that cause oxidative cleavage of the phosphate-sugar backbone and oxidation of the purine/pyrimidine residues of DNA [41]. The oxidation of catechol estrogen moieties generates estrogen semiquinones and quinones, namely estrogen-2,3-semiquinone and estrogen-3,4-semiquinone [37]. In addition to oxidative damage to DNA by ROS, estrogenic quinones can lead to the formation of mutagenic depurinating adenine and guanine adducts (4-OHE2-1-N3Ade and 4-OHE2-1-N7Gua) [47]. DNA adduct formation and depurination at critical locations such as tumor suppressors or oncogenes can lead to mutations that have been hypothesized to be the origin of cancer [37]. In addition to causing oxidant-induced DNA damage, estrogens also cause lipid peroxidation and oxygen radical-mediated oxidation of protein amino acid residues to carbonyl-containing moieties [37]. Although there is no evidence to suggest that either 2-OH-E2 or 2-OH-E1 is carcinogenic [9], both have the ability to generate ROS and undergo metabolic redox cycling, similar to 4-OH-E2 [27]. On the basis of the available data, the 4-OH-E2/2-MeO-E2 ratio is significantly higher in patients with endometrial cancer than in controls [13]. This finding suggests that the 2-OHCEs/4-OHCEs ratio may be an important indicator of cancer risk. The absence of carcinogenic properties of 2-hydroxylated estrogen metabolites can potentially be attributed to their prompt deactivation through O-methylation mediated by COMT [48], their swift elimination from the body, and their relatively low estrogenic hormonal activity in comparison with 4-OH-E2 [37]. COMT catalyzes the O-methylation of 2-OH-E2 or 2-OH-E1 at the 2-OH and 3-OH locations, as well as the regulation of estrogen levels in the body, and it helps to eliminate potentially genotoxic quinone metabolites [47]. The biological impacts of the methylated and nonmethylated forms are distinct. Research has shown that, after being methylated, 2-hydroxyestrone has antiestrogenic properties [49]. The primary outcome of COMT-mediated O-methylation of 2-OH-E2 is 2-MeO-E2 [13]. This particular compound exhibits distinctive antitumorigenic properties [37]. Although most healthy cells are unaffected by 2-MeO-E2, it has a significant cytotoxic effect on a variety of malignant cells [43]. However, the mechanism by which 2-MeO-E2 protects against cancer is still not fully understood, it has been demonstrated that 2-MeO-E2 induces apoptosis in ovarian cancer cells by activating both the intrinsic and extrinsic apoptotic pathways [43]. It has been proven that it generates ROS and reactive nitrogen species, leading to nitro-oxidative stress and resulting in cell cycle arrest or cell death [9, 50]. In addition, 2-MeO-E2 leads to the phosphorylation of Bcl-2 and Bcl-xL and reverses their antiapoptotic effects. Moreover, it has been demonstrated that 2-MeO-E2 activates Bak, BAX, and mitochondria-dependent caspases in addition to increasing BAX levels and decreasing Bcl-2 concentrations [50]. These growth-inhibiting effects have been attributed to the effect of 2-MeO-E2 effect on tubulin polymerization. Microtubule destabilization also results in cell cycle arrest [22]. 2-MeO-E2 also induces cell cycle arrest during the G2/M phase. It causes the downregulation of cyclin B1 and phosphorylated Cdc-2 and the upregulation of p21WAF1/Cip1 in endometrial cancer cells, which correlates with G2/M arrest and p53 activation [43]. 2-MeO-E2 has antiangiogenic properties that mediate anticarcinogenic activity [47]. Researchers hypothesize that this metabolite suppresses angiogenesis through two different mechanisms: a direct effect through an inhibition of endothelial cell function and an indirect effect through the inhibition of the expression, nuclear accumulation, and transcriptional activity of HIF-1, which results in the suppression of vascular endothelial growth factor [43]. However, the role of 2-MeO-E2 in endometrial cancer remains unclear. Several studies have suggested that decreased levels of 2-MeO-E2 may be associated with an increased risk of endometrial cancer, whereas others have shown no significant relationship. The study, which included a sample of 179 patients and 336 controls, did not provide evidence in favor of the hypothesis suggesting that the 2-hydroxyestrogen pathway offers protection whereas the 16a-hydroxyestrogen pathway poses harm in hormone-dependent cancers. The findings revealed that levels of estrogen metabolites were significantly elevated in patients compared with the controls. However, there was no statistically significant difference observed in the 2-OHE1:16a-OHE1 ratio between cases and controls [36]. Further research is needed to determine whether 2-MeO-E2 can be used as a biomarker for endometrial cancer and to elucidate its potential role in the development and progression of this disease. However, studies on the level of oxidative stress in endometrial cancer patients are rare. Research analyzing inflammatory and oxidative stress markers in 37 patients with endometrial cancer showed that the levels of serum oxidative stress markers, including oxidized low density lipoprotein, nitric oxide, advanced glycation end-products, advanced oxidation protein products, and malondialdehyde were increased in all patients with endometrial carcinoma compared with healthy controls (*p* < 0.05). The levels of ferric reducing ability of plasma, an antioxidant marker, were lower in patients with cancer than in healthy controls (*p* < 0.05) [14].

### Biomarkers

The National Cancer Institute defines a biomarker as a “biological substance in body fluids or tissues that is indicative of a normal or abnormal process or of a condition or disease” [51]. Biomarker-potential substances can include endogenous metabolites, genetic products, peptides, proteins, and antibodies [8]. Before potential diagnostic biomarkers can be used in the clinical context, various obstacles must be overcome, including discovery, validation, and verification. Any innovative test must be carefully assessed in terms of its analytical performance, clinical validity, and clinical value.

All estrogens circulate in a wide range of concentrations, which can be extremely low in some patient cohorts. The exact levels of most particular estrogen metabolites in circulation are unknown and understudied. It is presumed that the levels of bioactive substances are lower than those of the predominant circulating estrogens [29]. As a result, there is a need for more sensitive and specific methods for measuring estrogen levels. In endometrial cancer metabolomic investigations, liquid chromatography-mass spectrometry (LC-MS), nuclear magnetic resonance spectroscopy (NMR) analytical techniques and immunoassays are commonly used [2]. Immunoassays have long served as the standard method for steroid analysis. It is simple to assemble them. On the other hand the immunoassay method is characterized by its lengthy duration and necessity for a greater sample volume [52]. Each immunoassay can measure only a single analyte. Furthermore, it is common for antibodies to exhibit cross-reactivity, leading to the generation of inaccurate outcomes. Particularly with direct assays that lack purification stages, care must be taken to avoid specificity issues [53]. NMR is a nondestructive technique that allows multiple measurements to be taken on the same sample, which can reduce the cost and time required for analysis [2]. With minimal sample preparation, NMR can generate qualitative values for both known and unidentified substances. This enables biomedical samples to be minimally altered before analysis, preventing bias and physiologically irrelevant perturbations. Despite these advantages, NMR has several of drawbacks for analyzing complex biofluids, including decreased sensitivity and a restricted dynamic range [54]. In contrast to NMR, LC-MS is highly sensitive and can be performed on small clinical samples with low molar amounts of analytes, which is advantageous for large-scale human investigations [2]. This technique has revolutionized the field of bioanalysis due to its high selectivity, sensitivity, and speed. Mass spectrometry-based techniques offer valuable insights into the structural characteristics of the analytes, thereby enhancing their specificity. When used in conjunction with chromatographic methods, these techniques enable the concurrent determination of numerous analytes. Gas chromatography-mass spectrometry represents the most effective approach for characterizing steroid metabolomes because it has the highest level of specificity. This technique, although intricate, is widely recognized as a powerful tool in the field. LC-MS is a widely employed analytical technique known for its ability to process large volumes of samples efficiently. This method is particularly well-suited for the detection and analysis of intricate steroid compounds. Gas chromatography-mass spectrometry and LC-MS are not in direct competition with each other, but rather serve as complementary analytical techniques [44]. The stable isotope dilution methodology coupled with liquid chromatography-tandem mass spectrometry (LC-MS/MS) is quickly becoming the gold standard for measuring estrogens in serum and plasma due to its increased specificity, high accuracy, and capacity for a more comprehensive analysis [38]. As a result, biofluid-based biomarker discovery has become an active area of research with promising applications in personalized medicine [13]. It is important to consider confounding variables when developing and validating biomarkers for endometrial cancer. Usually, possible indicators are selected based on tumor biology. It has been proven that demographic variability (such as age, body mass index, blood pressure, and diabetes) and variability from exogenous sources of metabolites are significant confounders in biomarker research [55]. Therefore, future studies should focus on identifying and controlling for these confounding variables to improve the accuracy and reliability of the use of endogenous estrogen metabolites as biomarkers for endometrial cancer. Recent research has suggested that the local E2 metabolism, in particular, can be of considerable biological significance. Such local changes can be detected via measurements of samples from tissues, blood, or urine [49]. Examples of metabolites whose concentrations were determined and considered markers of endometrial cancer there are presented in Table 1.

**Table 1:** Potential biomarkers for endometrial cancer in biofluids

Estrogen Metabolite as Potential Biomarker	Biofluids	Level in the Endometrial Cancer Cases Compared with the Controls	References
Estrone	Serum	↑ or ↔	*13,16,17,21,36*
Estradiol	Serum	↑	*13,16,17,21,36*
	Urine	↑	*14*
2-Hydroxyestrone	Serum	↑	*36, 37*
	Urine	↔	*14*
2-Hydroxyestradiol	Serum	↑ or ↔	*36, 37*
	Urine	↔	*14*
2-Methoxyestrone	Serum	↔	*36, 37*
	Urine	↓	*14*
2-Methoxyestradiol	Serum	↔	*36, 37*
	Urine	↓	*14*
4-Hydroxyestrone	Serum	↑	*36, 37*
	Urine	↔	*14*
4-Hydroxyestradiol	Serum	↑	*36, 37*
	Urine	↑	*14*
4-Methoxyestrone	Serum	↔	*36, 37*
4-Methoxyestradiol	Serum	↔	*36, 37*
16alpha-Hydroxyestrone	Serum	↑	*36, 38*
	Urine	↔	*14*
Estriol	Serum	↑ or ↔	*37*

↑significant increase ↓significant decrease ↔ insignificant difference

### Blood-based biomarkers

Because blood is easily available and its usage is generally acceptable to both clinicians and patients, blood is a good source of biomarkers. The optimal management of patients is contingent upon the accuracy and validity of E2 metabolite assays; therefore, clinicians must consider analytic interference and the fact that the laboratory’s accuracy is not reliable. False elevation of the serum E2 concentration due to analytical interference is uncommon and is typically associated with cross-reactive substances, such as the aromatase inhibitor exemestane and the selective ER degrader fulvestrant [56]. Falsely elevated levels of a supposed biomarker can lead to needless investigations and interventions, as well as severe mental stress. On the other hand, human blood contains relatively small amounts of catechol estrogen metabolites. This may result in false-negative results, which may cause missed or delayed diagnosis. New developments in metabolomics have made it possible to identify and quantify a wide variety of metabolites. Limits of quantitation by LC-MS/MS for E1 and E2 over a broad range of 0.14–3000 pg/mL have been reported [29]. Incorporating metabolites into assay palettes may be associated with a loss of sensitivity. Usually, 0.1–2 mL of serum or plasma is needed, but approximately 0.5 mL is preferred for routine collection [29]. A case-cohort study was conducted to evaluate the associations between 15 prediagnostic serum estrogens and estrogen metabolites and the risk of incident endometrial cancer in postmenopausal women who were not receiving hormone replacement therapy. Sixty-six patients with endometrial cancer participated in this study. LC-MS/MS was used to measure the concentration of serum estrogens and estrogen metabolites. The concentrations of catechol estrogens, as well as 16-hydroxyestrone, in the 2- and 4-hydroxylation pathways, were substantially greater than those in the controls (*p* < 0.05) [34]. These findings suggest a correlation between increased serum levels of catechol estrogens and 16-hydroxyestrone and the incidence of endometrial cancer in postmenopausal women. A case-control study, conducted in 93,676 postmenopausal women, revealed 15 estrogens/estrogen metabolites in patients with overall and subtype-specific endometrial cancer [35]. The most abundant estrogen was conjugated E1, followed by estriol. Several metabolites, including metabolites of the 4-hydroxylation pathway, and 2-methoxymetabolites, were detected at quite low levels. All the parent estrogens and individual metabolites were found in higher concentrations in patients with endometrial cancer than in controls. This study also revealed the biology of the tumor as a confounding factor. There was significant heterogeneity in the associations of several estrogens between the two tumor types, including unconjugated E2 (the odds ratio for the highest vs. lowest quintile for type I tumors was 6.97 (95% CI, 3.20–15.20) vs. 1.80 [0.43–7.49] for type II tumors). Other significant differences were detected for unconjugated 2-methoxyestrone (3.47 vs. 0.95) and 4-methoxyestrone (3.54 vs. 0.84) [35]. Nonetheless, these studies provide valuable insight into the potential role of estrogen metabolism in endometrial cancer and highlight the need for further investigation in this area.

### Urine-based Metabolite biomarkers

An alternative source of samples is urine, which can be collected easily, usually with no limit. Its collection is inexpensive and typically free of problems or adverse effects [8]. Urinary metabolites may be generated from chemically changed systemic metabolites that are expelled in the urine or from contamination of the urine by tumor-derived metabolites that are deposited in the lower genital tract [13]. Research comparing urine samples desiccated on filter paper and analyzed by gas chromatography with tandem mass spectrometry with serum samples analyzed by conventional radioimmunoassay, has shown high degree of reliability [57]. By analyzing small volume samples of urine using mass spectrometry, it was possible to obtain a comprehensive hormone profile, allowing for an expanded view of both hormone production and clearance [58]. A highly validated ultra-performanceLC-MS/MS analytical method, characterized by its rapidity and sensitivity, was employed for the quantification of 13 estrogens in human urine. The specificity, linearity, intra- and interday precision and accuracy, recovery and matrix effect, stability and dilution effect of the ultra-performanceLC-MS/MS method met the requirements for the analysis of biological samples for methodological validation by the Food and Durg Administraton [52]. To assess the absolute concentrations of endogenous estrogens and their metabolites, 0.5 mL of urine was needed for the procedure [59]. The results obtained from dried urine were strongly coherent with those obtained from liquid urine [58]. This noninvasive method could be used for early detection of endometrial cancer. However the evaluation of urinary hormone metabolites can be used instead of serum hormone analysis, and critical factors influence the results obtained. The timing of sample collection is crucial because urine testing offers information over a range of times and with a lag caused by metabolic processing, whereas serum testing provides status at the time of collection [57]. Even thoughthat metabolomic analysis of urine has high potential to detect clinically meaningful biomarkers, this is achievable only when environmental factors are under control. Using urine samples from 23 endometrial cancer cases and 23 postmenopausal healthy controls, Zhao et al. identified endogenous estrogen metabolites as biomarker candidates for endometrial cancer. High-performance LC-MS was used to assess the presence of two estrogens and their six metabolites in the urine samples. In the present study, the E2/E1 ratio in the case group was greater than that in the controls (*p* < 0.01). When 4-OH-E2 was increased, 2-MeO-E1 and 2-MeO-E2 were low in the urine samples patients with endometrial cancer (*p* < 0.01, *p* < 0.01). Urinary E2 levels were also observed to be higher in endometrial cancer cases. In the investigation of patients with endometrial cancer, the concentrations of 16-OH-E1, 2-OH-E2, and 2-OH-E1 were not correlated with the incidence of cancer [13].

### Saliva-based metabolite biomarkers

As an alternative to serum and urine, saliva is readily available, safe to handle and painless and easy to collect, and its collection is generally accepted. Passage through the lipophilic layers of the capillaries and glandular epithelial cells regulates the rate at which hormones are transferred from the blood to saliva. Therefore, lipophilic molecules, such as steroids, pass through these barriers faster than hydrophilic molecules, such as peptides [60]. Salivary steroid hormone concentration assessments are convenient for quantifying the physiologically active hormones because they are strongly correlated with the free, unbound hormone fraction found in the blood [61]. Currently, most studies use a device that is inserted either under the tongue or in the cheek to capture saliva, which is then centrifuged and extracted. The enzyme-linked immunoassay and radioimmunoassay techniques are commonly used [47]. These techniques are mainly chosen due to their low cost, simplicity of use, and high sample throughput [53]. Because these techniques rely on the properties of the antibody used and frequently show cross reactivity between distinct estrogens of interest and other species, they can have poor selectivity [53]. This issue is especially noticeable when measuring low levels [47]. Most endometrial cancer diagnoses occur in postmenopausal women between the ages of 55 and 64 [62]. Some data suggest that salivary E2 measurements are an adequate estimation of serum E2 in postmenopausal women, but only when estrogen replacement therapy is administered. In postmenopausal women who do not use estrogen replacement therapy, estradiol levels in saliva samples may be too low for accurate measurement [63]. For accurate analysis of E1 and E2, particularly in postmenopausal women, high selectivity at low concentrations is essential, and the same rigor is required to assay low levels of bioactive estrogen metabolites. Consequently, the development of robust analytical methodologies capable of simultaneous estrogen quantification at low circulating concentrations is required. Recently, the application of online solid phase extraction methods has resulted in an increase in method sensitivity and a reduction in pretreatment and analysis timeframes. The combination of online solid phase extraction and LC-MS/MS methodology may therefore provide a highly specific and sensitive analytical strategy for the quantification of endogenous salivary steroids that is applicable in an applied or clinical research area [61]. Therefore, saliva-based metabolite biomarkers can be a useful tools for the early detection and monitoring of endometrial cancer, as they provide a noninvasive and convenient method for analyzing hormone levels in patients. However, further studies are needed to validate the accuracy and reliability of these biomarkers.

## Summary

Endometrial cancer is a common gynecological malignancy that affects women worldwide. Despite advances in treatment, the prognosis for patients with advanced disease remains poor. Early detection is crucial for improving treatment outcomes, but current screening methods are limited in their sensitivity and specificity. Identifying biomarkers that can accelerate and facilitate the detection of endometrial cancer in low- or noninvasive samples such as blood, urine, or saliva could revolutionize the way we diagnose and treat this disease. By identifying biomarkers that are specific to endometrial cancer, including its subtypes, we can improve early detection rates and personalize treatment. Estrogen plays a key role in the development of endometrial cancer, and its metabolites have been shown to be altered during the course of this disease. Available data suggest that biomarkers based on estrogen metabolites from different pathways could be useful for identifying women at high risk for this disease. However, further research is needed to fully understand the complex interplay between estrogen metabolism and endometrial cancer development. Our research team is already analyzing the research results in biofluids.

## Data Availability

No datasets were generated or analysed during the current study.

## Abbreviations

ROS: reactive oxygen species
RNS: reactive nitrogen species
EC: endometrial cancer
PMB: postmenopausal bleeding
TVUS: transvaginal ultrasonography
ET: endometrial thickness
E2: estradiol
E1: estrone
HSD17: 17-hydroxysteroid dehydrogenase
2-OH-E2: 2-hydroxyestradiol
2-OH-E1: 2-hydroxyestrone
4-OH-E2: 4-hydroxyestradiol
4-OH-E1: 4-hydroxyestrone
16-OH-E1: 16-hydroxyestrone
2-MeO-E1: 2-methoxyestrone
4-MeO-E1: 4-methoxyestrone
2-MeO-E1: 2-methoxyestradiol
4-MeO-E2: 4-methoxyestradiol
COMT: catechol–O–methyltransferase
ERs: estrogen receptors
LC-MS: liquid chromatography–mass spectrometry
NMR: nuclear magnetic resonance spectroscopy
GC-MC: gas chromatography–mass spectrometry
RIA: radioimmunoassay
